# Diosmetin Mitigates Cognitive and Memory Impairment Provoked by Chronic Unpredictable Mild Stress in Mice

**DOI:** 10.1155/2020/5725361

**Published:** 2020-12-21

**Authors:** Elham Saghaei, Shakiba Nasiri Boroujeni, Parvin Safavi, Zeinab Borjian Boroujeni, Elham Bijad

**Affiliations:** ^1^Department of Physiology and Pharmacology, Faculty of Medicine, Shahrekord University of Medical Sciences, Shahrekord, Iran; ^2^Medical Plants Research Center, Basic Health Sciences Institute, Shahrekord University of Medical Sciences, Shahrekord, Iran; ^3^Department of Psychiatry, Faculty of Medicine, Shahrekord University of Medical Sciences, Shahrekord, Iran; ^4^Student Research Committee, Shahrekord University of Medical Sciences, Shahrekord, Iran

## Abstract

**Materials and Methods:**

In the present experimental study, male NMRI mice were exposed to chronic unpredictable mild stress (CUMS) paradigm for 35 days. Diosmetin (at doses of 10, 20, and 40 mg/kg. i.p.) or diosmetin solvent (normal saline + DMSO, 1 ml/kg; i.p.) was administered 30 min before stress induction. After 28 days, memory and cognitive performance were assessed by shuttle box and novel object recognition tests. Finally, antioxidant capacity (FRAP) and malondialdehyde (MDA) level of serum and brain, and serum corticosterone level were evaluated.

**Results:**

Behavioral tests showed that CUMS significantly reduced the secondary latency in passive avoidance memory test and diagnosis index in novel object recognition test compared to the control group (*P* < 0.001), whereas treatment with diosmetin (20 and 40 mg/kg) significantly improved memory performance in the two tests (*P* < 0.001). In addition, diosmetin (40 mg/kg) could pronouncedly suppress increase in serum corticosterone levels, reduction in antioxidant capacity, and production of excess MDA caused by CUMS compared to the control group (*P* < 0.01, *P* < 0.001, and *P* < 0.001, respectively).

**Conclusion:**

Chronic stress can impair memory and cognition and treatment with diosmetin can partly improve this disorder in male mice by increasing the antioxidant capacity of brain tissue and serum and improving serum corticosterone levels.

## 1. Introduction

Memory is a process by which acquired information is stored and re-read through learning. Learning is the process of acquiring new information from the surrounding world while memory refers to the ability to memorize and retrieve it [1]. In the cerebral cortex, there are primary and secondary motor areas, primary and secondary sensory areas, and special areas for visual, auditory, and physical sensations. In addition, there are other cortical areas in the cortex called communication areas, which receive and analyze signals from multiple areas of the motor and sensory cortex, and subcutaneous structures. Synapses that are subjected to repetitive presynaptic nerve stimulation cause changes in the excitability of postsynaptic neurons. These changes include the facilitation of neuron activation, changes in the pattern of neurotransmitter release, and secondary messenger formation, which eventually lead to learning [2]. Synaptic facilitation, long-term potentiation (LTP), and long-term depression (LTD) are the most important mechanisms involved in memory formation [2].

Studies have shown that chronic stress can block chemical reactions in the brain that are useful for learning and memory. Chronic stress can also impair growth, learning, and memory [3]. Chronic stress induces its effect by activating the hypothalamic-pituitary-adrenal (HPA) axis and increasing glucocorticoid levels. Glucocorticoids can have short- and long-term effects on cognitive behavior and processes through genomic and nongenomic mechanisms. Evidence suggests that long-term exposure to stress or glucocorticoids causes numerous changes in the structure of the hippocampus, including neurochemical alteration, changes in irritability, neurogenesis, neuronal morphology, and even cell death. Such changes in the hippocampus have been proposed as the underlying mechanism of stress-induced cognitive impairments [4].

Treatments may include anxiolytics such as benzodiazepines (lorazepam, diazepam), acetylcholinesterase inhibitors (rivastigmine or donepezil) that are also used for the treatment of Alzheimer's disease, or antidepressants (fluoxetine, imipramine, etc.). These treatments address some aspects of the disorder that do not necessarily lead to appropriate responses and have numerous side effects. In seeking out new therapeutic products for the treatment of neurodegenerative disorders with fewer side effects, research across the world has consistently shown the efficacy of different types of herbal remedies in various animal models [5–7].

**Figure 1:**
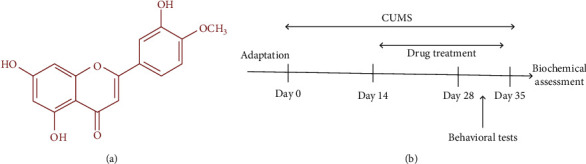
The molecular structure of diosmetin (A). A schematic paradigm of CUMS procedure and drug treatment (B).

Diosmetin (5,  7,  3′, -trihydroxy-4′,-methoxyflavone, Figure 1(a)) is a methylated flavone which is naturally found in the citrus family and in plants of the genus Lamiaceae, *Teucrium*, Portuguese, and olive leaves [8, 9]. Pharmacologically, diosmetin has been reported to exhibit anti-cancer, antimicrobial, antioxidant, and anti-inflammatory, phytoestrogenic, and osteogenic activity [8, 9]. Diosmetin also acts as an antioxidant receptor agonist for tropomyosin receptor kinase B (TrkB) [10]. Diosmetin exhibits neuroprotective effects and increases serum antioxidant capacity in vitro due to its chemical structure that is similar to that of catechin and quercetin flavonoids, and by converting it to the intermediate compound luteolin [11, 12].

Antioxidant effects of diosmetin have been reported to be induced by inhibition of nitric oxide (NO) production and by increasing the serum antioxidant capacity of animals given this compound [13]. Diosmetin also enhances antioxidant activity in monocytes by inhibiting the production of intracellular reactive oxygen species [14] and malondialdehyde (MDA), and enhancing the intracellular antioxidant effects of superoxide dismutase (SOD) and catalase and glutathione peroxidase (GPx) [15]. This compound produces its protective effects by reducing oxidative stress markers and preventing oxidative damage to the retinal DNA on apoptosis of adriamycin-induced retinal granule epithelial cells [16]. The anti-inflammatory properties of diosmetin have been demonstrated through reduced nitric oxide production and decreased secretion of tumor necrosis factor-alpha (TNF-*α*) from microglia and macrophages in vitro [15]. Diosmetin can suppress the apoptosis of T48 cells by activating the phosphorylation of AKt and ERK protein kinases [17].

Given the negative effects of chronic stress on cognition and memory and the numerous protective effects of diosmetin, this study investigated the effect of diosmetin on cognitive impairment and memory due to chronic stress.

## 2. Materials and Methods

### 2.1. Animals

A total of 80 adult NMRI mice were selected in the weight range of 20–30 g and kept in appropriate conditions (21 ± 2)°C, 12-hour light and 12-hour dark with free access to water and food.

All procedures of this study were conducted according to the regulations of the University and the Guide for the Care and Use of Laboratory Animals of National Institutes of Health (ethics number: IR.SKUMS.REC.1395.157) and Guide for the Care and Use of Laboratory Animals (Eighth Edition, 2011, published by the National Press).

### 2.2. Chronic Unpredictable Mild Stress (CUMS) Procedure and Experimental Design

CUMS was induced by a method described by Willner with minor modification [18]. The paradigm included separate stressors, which were randomly set up. All stressors are listed in Table 1. Animals were divided into CUMS and normal groups. In CUMS groups, animals were exposed to stressors for 2 weeks and then distributed into 4 groups (*n* = 10 per group) and received solvent (saline + 2% DMSO) and/or diosmetin (Sigma Aldrich, United Kingdom, D7321) (10, 20, 40 mg/kg) for 3 weeks while being exposed to CUMS. Besides, normal animals were divided into 4 groups and received normal saline or diosmetin (10, 20, 40 mg/kg) for 3 weeks (Figure 1(b)).

It should be noted that the injections of the drugs were conducted intraperitoneally and the drugs dosages were selected based on previous studies [19].

After completion of the behavioral test, the animals were anesthetized with ketamine and xylazine (60 and 10 mg/kg, respectively) and blood samples and the hippocampus tissues were isolated and immediately transferred to the freezer set at −70°C.

### 2.3. Cognitive Function and Memory Test through Novel Object

The novel object recognition test is a method to test the cognitive function and memory of the animal. The test was done according to the method of Bertaina et al. In summary, a black wooden box of 40 × 40 × 40 cm was used. Animals were placed in the apparatus on the day before the test to adapt to it. After 24 hours, two identical objects were placed in two corners of the box, allowing the animal to identify and examine the objects for 5 min. The animal was then removed from the box and 30 minutes later, the test was performed in which one of the objects changed and the length of time the animal spent identifying each object was recorded [20]. This method consists of three stages of habituation, learning (T1), and memory testing (T2). In this method, the difference between the time the animal spends identifying an old object (T1) and a new object (T2) is used as the criterion to evaluate memory, known as the recognition index or discrimination index.

Recognition index = *T*2/(*T*1+T2).

### 2.4. Passive Avoidance Memory Test through Shuttle Box

The shuttle box includes an electric shocker and two light and dark chambers separated by a guillotine door. The bottom of the apparatus is made of a conductive metal grid. In the first and second days of the test, the animal is allowed to adapt to the apparatus by allowing it to move freely between the light and dark chambers for 5 minutes. On the third day, when the mouse was released into the bright room, 20 seconds later the guillotine door was removed and the time of entry into the dark room was recorded by a stopwatch. When the animal entered the dark chamber, the animal was given an electric shock (1 mA, 1 sec, 1 time) and then the animal was ejected. On the fourth day, the time interval between being in the bright room and entering the dark room was measured and expressed as the delay time (to a maximum of 60 sec) [21].

### 2.5. Serum Corticosterone Determination

Serum corticosterone levels were measured by specific mouse corticosterone ELISA kit (Abcam, United Kingdom, ab108821). All reagents and samples were prepared according to the kit instruction. The absorbance of samples was assayed on microplate ELISA reader at a wavelength of 450 nm. Results were reported in ng/ml [22].

### 2.6. Antioxidant Capacity Determination

The total antioxidant capacity of serum and brain tissue was assessed by the FRAP method described by Benzie, based on the ability of serum and tissue sample to recover ferric ions to ferrous ions in the presence of 2,4,6-tripyridyl-*s*-triazine (TPTZ), and the absorbance was recorded by a spectrophotometer. Briefly, 50 *μ*l of 10% homogenate suspension extract or serum was added to 1.5 ml of freshly prepared reactant (including acetate buffer solution with pH 3.6, TPTZ, HCl 20 mM, and FeCl_3_ 20 mM) and incubated at 37°C for 10 min [23]. The intensity of blue color formed by the complex between ferrous ion and TPTZ was measured by a spectrophotometer at 593 nm. All data were reported in *μ*M [21, 24].

### 2.7. MDA Level Determination

The MDA levels of hippocampus and serum were measured according to the method described by Zarrindast et al. (1991). Briefly, 1 g of homogenized hippocampus (using 2.5% potassium chloride) sample or 1 ml of serum one was added to reaction solution containing 1 ml of 5% trichloroacetic acid and 1 ml of 67% thiobarbituric acid and then centrifuged at 2000 g for 15 min. The supernatant was then transferred to another tube and placed in a boiling bath for 10 min. After cooling down, the absorbance of the prepared sample was measured by a spectrophotometer at 535 nm, and the results were expressed as *μ*g/ml [25].

## 3. Data Analysis

Statistical analysis was performed using SPSS 16 software. First, data were analyzed using Kolmogorov–Smirnov test and then homogeneity of variance was estimated using Levene's test. One-way ANOVA was used to determine the significant differences between treatments and Tukey's post hoc test was used to compare the mean values. Data were recorded as mean ± standard error and *P* < 0.05 was considered as the significance level.

## 4. Results

### 4.1. Diosmetin Increased the Secondary Latency of Passive Avoidance Memory

**Figure 2:**
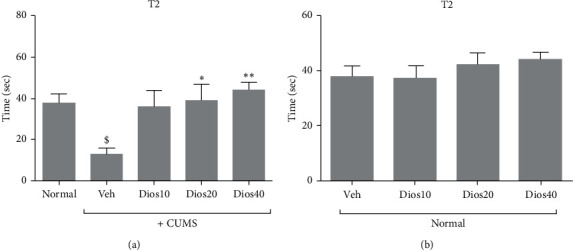
The secondary latency (T2) on passive avoidance memory in shuttle box test in CUMS conditions (A) and in nonstressful conditions (B). Normal: healthy group. Veh: diosmetin solvent received group. Dios: diosmetin-receiving group (10, 20, 40 mg/kg). $*P* < 0.05 compared to the normal group. ^*∗*^*P* < 0.05 and ^*∗∗*^*P* < 0.01 compared to the vehicle treated group.

According to the data regarding the shuttle box test (Supplementary Table S1 and Figure 2), the rate of secondary delay in the CUMS group was significantly reduced compared to the healthy group (*P* < 0.05). Different doses of diosmetin in a dose-dependent manner increased the secondary latency in CUMS animals compared to the solvent-receiving stress group. However, significant differences were observed only in the groups receiving doses of 20 and 40 mg/kg (*P* < 0.05 and *P* < 0.01, respectively). In the stress-free condition at doses of 20 and 40 mg/kg diosmetin, the delay time increased, but the increase in the secondary delay time was not significant compared to the solvent-receiving group.

### 4.2. Diosmetin Improved Memory Parameters in the Novel Object Test

**Figure 3:**
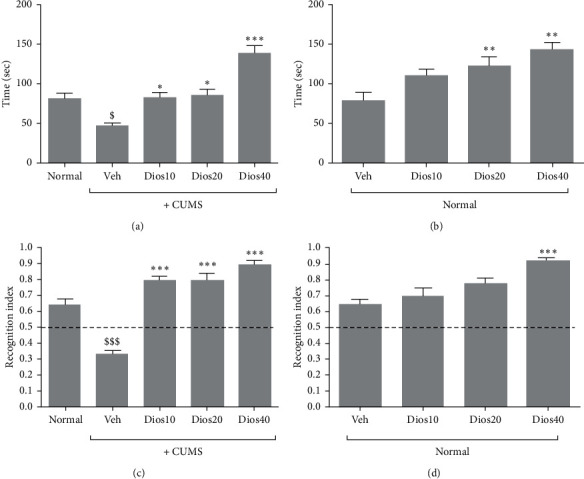
The duration of new object recognition (T2) in CUMS condition (A) and in stress-free condition (B). The recognition index in CUMS condition (C) and in stress-free condition (D) in the novel object test. Normal: healthy group. Veh: diosmetin solvent-receiving group. Dios: diosmetin-receiving group (10, 20, 40 mg/kg). $*P* < 0.05 and $$$*P* < 0.001 compared to the normal group. ^*∗*^*P* < 0.05, ^*∗∗*^*P* < 0.01, and ^*∗∗∗*^*P* < 0.001 compared to the vehicle-treated group.

The results (Figures 3(a)–3(d), and Supplementary Table S2) revealed that the duration of new object recognition test decreased significantly in the CUMS group compared to the healthy group (*P* < 0.05). Diosmetin increased T2 dose-dependently in the animals under chronic stress compared to the solvent-treated CUMS group, with the highest effect at 40 mg/kg (*P* < 0.001, Figure 3(a)).

Under stress-free conditions (Figure 3(b)), the duration of new object recognition dose-dependently increased. In the groups given diosmetin at doses of 20 and 40 mg/kg, new object recognition time significantly increased in comparison to the solvent-receiving group (*P* < 0.05).

According to the results (Figure 3(c)), the recognition index in the CUMS group showed a significant decrease compared to the normal group (*P* < 0.001). In the diosmetin-treated CUMS groups, the recognition index increased by more than 50% and was proportional to the increase in diosmetin dose, and a significant difference was observed in the chronic stress group receiving solvent (*P* < 0.001).

In nonstress conditions (Figure 3(d)), the results revealed a significant difference in the recognition index only in the group receiving 40 mg/kg diosmetin compared to the solvent-receiving group (*P* < 0.001).

### 4.3. Diosmetin Reduced Serum Corticosterone Levels

**Figure 4:**
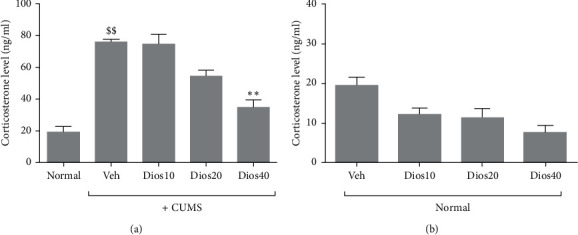
Serum corticosterone levels in CUMS condition (A) and stress-free conditions (B). Normal: healthy group. Veh: diosmetin solvent-receiving group. Dios: diosmetin-receiving group (10, 20, 40 mg/kg). $$*P* < 0.01 compared to the normal group. ^*∗∗*^*P* < 0.01 compared to the vehicle-treated group.

Corticosterone levels in the CUMS group showed a significant increase compared to the healthy group (*P* < 0.01, Figure 4(a)). Moreover, serum corticosterone levels decreased in the diosmetin-treated chronic stress groups. However, there was a significant difference in corticosterone levels between the group given 40 mg/kg of diosmetin and the solvent-receiving group under chronic unpredictable mild stress (*P* < 0.01). Corticosterone levels in stress-free condition (Figure 4(b)) decreased following injection of different doses of diosmetin in a dose-dependent manner, but the difference between the solvent group and the diosmetin group was not significant. Detailed data regarding the serum corticosterone levels of different groups are presented in Supplementary Table S3.

### 4.4. Diosmetin Reduced the Antioxidant Capacity of Brain Tissue and Serum

**Figure 5:**
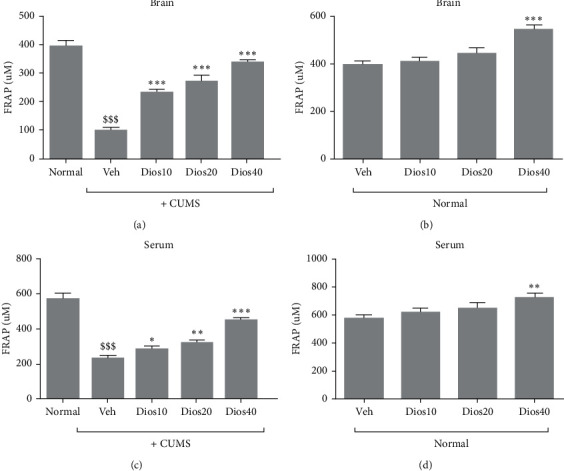
Antioxidant capacity of brain tissue in CUMS (A) and under stress-free conditions (B) and serum antioxidant capacity CUMS conditions (C) and stress-free conditions (D). Normal: healthy group. Veh: diosmetin solvent-receiving group. Dios: diosmetin-receiving group (10, 20, 40 mg/kg). $$$*P* < 0.001 compared to the normal group. ^*∗*^(*P*) < 0.05, ^*∗∗*^*P* < 0.01, and ^*∗∗∗*^*P* < 0.001 compared to the vehicle-treated group.

The antioxidant capacity of brain tissue showed a significant decrease in the CUMS group compared to the healthy group (*P* < 0.001, Figure 5(a)). The results also showed that different doses of diosmetin, in a dose-dependent manner, significantly maintained the antioxidant capacity of brain tissue in the animals under CUMS compared to the solvent receiving group (*P* < 0.001).

In nonstress conditions (Figure 5(b)), the antioxidant capacity of brain tissue following injection of different doses of diosmetin showed a dose-dependent increase, with a significant difference only at 40 mg/kg compared to the solvent-receiving group (*P* < 0.001).

The results of serum antioxidant capacity (Figure 5(c)) showed a significant decrease in the CUMS group compared to the healthy group (*P* < 0.001). On the other hand, doses of 10, 20, and 40 mg/kg diosmetin significantly preserved serum antioxidant capacity in the chronic stress groups compared to the solvent-receiving group (*P* < 0.001 and *P* < 0.05, respectively).

In nonstress conditions (Figure 5(d)), the antioxidant capacity of the serum showed a dose-dependent increase after administration of different doses of diosmetin to the animals. Significant difference was observed only in the group that received diosmetin at the dose of 40 mg/kg compared to the solvent-receiving group (*P* < 0.001). The total antioxidant capacity datasets associated with the serum and hippocampal tissue of all groups are shown in Supplementary Table S4.

### 4.5. Diosmetin Reduced MDA Level in Hippocampal Tissue and Serum

**Figure 6:**
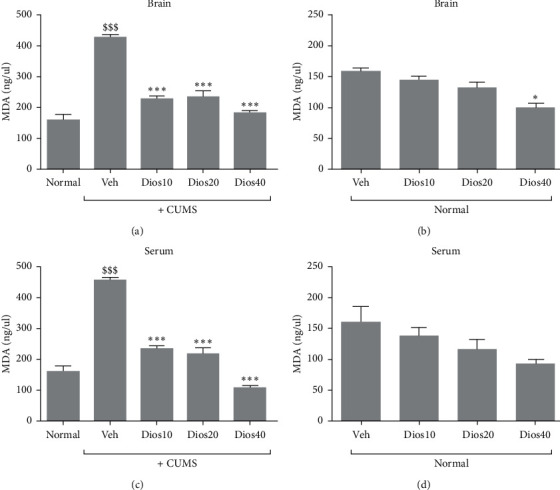
Evaluation of MDA level of hippocampal tissue in CUMS condition (A) and in stress-free condition (B) and serum MDA levels in CUMS condition (C) and in stress-free condition (D). Normal: healthy group. Veh: diosmetin solvent-receiving group. Dios: diosmetin-receiving group (10, 20, 40 mg/kg). $$$*P* < 0.001 compared to the normal group. ^*∗∗∗*^*P* < 0.001 compared to the vehicle-treated group.

As illustrated in Figure 6(a) and 6(c), the MDA levels of hippocampal tissue and serum significantly increased in the CUMS group compared to the normal group (*P* < 0.001). Additionally, our results revealed a dose-dependent and significant reduction in both serum and hippocampal tissue MDA levels in diosmetin-treated CUMS groups when compared to solvent-treated CUMS group (*P* < 0.001).

Effects of different doses of diosmetin on MDA concentration in normal animals are illustrated in Figure 6(b) and 6(d). Our results indicated that diosmetin at 40 mg/kg significantly reduced MDA level in hippocampal tissue compared to the solvent-treated normal group (Figure 6(b), *P* < 0.05). Additionally, no significant difference was observed in serum MDA levels between diosmetin-treated normal groups and solvent-treated normal group (Figure 6(d)). Supplementary Table S5 contains datasets related to the MDA levels of hippocampal tissue and serum.

## 5. Discussion

In the present study, the effect of diosmetin on memory impairment due to CUMS was investigated. The results showed that five weeks of CUMS impaired memory function. The symptoms were associated with increased hypothalamic-pituitary-adrenal (HPA) axis activity, decreased antioxidant capacity, and increased MDA production in the brain hippocampal tissue and serum. Diosmetin treatment significantly restored CUMS-induced behavioral changes. Moreover, diosmetin significantly maintained normal corticosterone levels, increased the antioxidant capacity, and decreased the MDA levels of hippocampal tissue and serum in animals under CUMS.

The results from the novel object recognition and shuttle box tests were similar to those of previous studies which showed that chronic stress can notably diminish memory and cognitive performance [26, 27]. According to the results from behavioral tests, in chronic passive avoidance memory (shuttle box), which reflects the animal's cognitive ability and recall of obtained information in avoiding the disturbing stimulus of electric shock, chronic stress impairs electrical shock avoidance. This test shows damage to the hippocampus and, to some extent, the amygdala. Inhibition of these two regions by drugs or their degradation may inhibit chronic stress memory impairment [28]. Furthermore, the novel object recognition test strongly represents visual cognitive memory and is considered as a short-term memory model [29, 30]. It has been shown that in chronic stress model, in addition to the hippocampus, the prefrontal cortex structure is also likely to be affected [14].

Diosmetin increased the speed of learning in chronic stress animals in the avoidance memory test and also significantly increased the time of avoiding electric shock and entering the dark room compared to the group under stress. Similarly, different doses of diosmetin significantly increased the recognition index in novel object recognition test by more than 50% in comparison with the group under stress. In the other studies, various flavonoid compounds such as diosmin (a flavone glycoside of diosmetin) [31], quercetin [32, 33], hesperetin [34], luteolin [35, 36], crocin [37], and nobiletin [38] *h* have been found to improve memory and learning performance in animals.

One of the important mechanisms involved in cognitive and memory impairment is the effect of chronic stress through elevation of glucocorticoid levels and activation of the HPA axis. In fact, it seems that chronic stress constantly stimulates the HPA axis, leading to glucocorticoids release. As a result, brain regions related to memory formation and consolidation, such as hippocampus and amygdala, undergo a variety of changes due to persistent exposure to glucocorticoids [4]. The corticosterone, as the main glucocorticoid in rodents, attenuates synaptic plasticity in the hippocampus, prefrontal cortex, and striatum, that accompany behavioral and cognitive impairment [39–42]. In the present study, it was found that the level of corticosterone significantly increased in the CUMS group in comparison with stress-free animals, which is consistent with the proven hypothesis mentioned above. The use of corticosterone synthesis inhibitors such as metyrapone can improve the stress-related cognitive impairment induced by corticosterone [40, 42]. Additionally, administration of diosmetin to animals under CUMS maintained a normal corticosterone level, which may have partly contributed to the reversal of memory deficit induced by chronic stress. Similarly, there have been various reports on the effect of flavonoid compounds, such as quercetin, luteolin, and crocin, on the reduction of corticosterone levels in stress conditions [22, 33, 43].

On the other hand, one of the notable results of this study was the nonsignificant reduction in corticosterone levels in diosmetin-treated normal groups when compared with saline-treated normal group. Unfortunately, there was insufficient data to support the current finding, whereby it is necessary to investigate the exact mechanism associated with this result because previous studies indicated that memory performance deficits correlate to not only very high but also very low levels of corticosterone [40, 42]. However, the memory performances of diosmetin-treated stress-free animals were not proportional to the corresponding reduced corticosterone levels.

Frequent exposure of neural cells to glucocorticoids due to chronic stress increases ROS and RNS production and the sensitivity of neurons to the deleterious effects of the latter compounds, which play a pivotal role in memory impairment [44]. In addition, oxidative stress and lipid peroxidation can damage memory through the mechanism of oxidative damage to the hippocampus and pyramidal cell apoptosis that reduces glucocorticoid receptors and increases glucocorticoid production [45]. In the present study, the results regarding antioxidant capacity and MDA production in chronic stress conditions were greatly consistent with previous studies.

One of the prominent properties of flavonoid compounds is their antioxidant effect that has been investigated in numerous studies on cognitive and memory impairment [22, 33, 46, 47]. The effect of diosmetin in preventing various cell damage due to oxidative stress and toxins has been demonstrated in vitro. In previous studies, diosmetin has been found to protect hepatocytes against cellular damage caused by iron accumulation and tert-butyl hydroperoxide toxin [10, 48]. Diosmetin has also exhibited therapeutic and protective effects in experimental models of various diseases such as retinal damage, oxidative damage to erythrocyte and serum induced by AAPH and CuCl2, acute pancreatitis caused by ceruline, and acute lung injury. These effects are produced due to the enzymatic and nonenzymatic effects of antioxidants (myeloperoxidase depletion, depletion of reactive oxygen species, depletion of antioxidant enzymes, and decrease in lipid peroxidation) and the reduction of expression of inflammatory factors and oxidative stress-regulating genes and proinflammatory cytokines (TNF-*α*, IL-1*β*, and IL-6) of this compound [10, 16, 19, 49]. In the present study, consistent with the results of previous studies, different doses of diosmetin significantly and dose-dependently preserved the antioxidant capacity and reduced MDA production in CUMS animals.

## 6. Conclusion

Taken together, according to the results of this study, some of memory-enhancing properties of diosmetin in CUMS condition might be associated with the efficacious regulation of oxidative stress through augmentation of total antioxidant capacity and decreasing of lipid peroxidation as well as preserving normal corticosterone. However, further studies are needed to elucidate the precise mechanism of how diosmetin affects memory deficit in chronic stress conditions.

## Figures and Tables

**Table 1:** The stressors of CUMS protocol.

Stressor	Duration (h)
Water deprivation	24
Food deprivation	24
Cage tilt (45°)	12
Physical restraint	6
Wet caging	12
Social isolation	24
Cold water swimming (4°C)	5
Overnight illumination	12
Soiled cage	12
Grouped housing	24
Continue lighting	24

## Data Availability

The data used to support the findings of this study are included both within the article and within the supplementary information file.

## Supplementary Materials

Supplementary Materials Supplementary Table S1: the datasets of shuttle box test. Supplementary Table S2: the datasets of novel object test. Supplementary Table S3: the datasets of serum corticosterone level. Supplementary Table S4: the datasets of antioxidant capacity. Supplementary Table S5: the datasets of malondialdehyde level.
