# Supplementary material for: Diosmetin Mitigates Cognitive and Memory Impairment Provoked by Chronic Unpredictable Mild Stress in Mice
Source: Evid Based Complement Alternat Med. 2020 Dec 21;2020:5725361. doi: 10.1155/2020/5725361 (PMC7769643; doi:10.1155/2020/5725361)
Supplement: Supplementary Materials — Supplementary Table S1: the datasets of shuttle box test. Supplementary Table S2: the datasets of novel object test. Supplementary Table S3: the datasets of serum corticosterone level. Supplementary Table S4: the datasets of antioxidant capacity. Supplementary Table S5: the datasets of malondialdehyde level. [file 5725361.f1.pdf]

**Supp. Table1.** The datasets of Shuttle Box test

**A**

| CUMS | | | | |
|---|---|---|---|---|
| **Normal** | **Veh** | **Dios10** | **Dios20** | **Dios40** |
| 34 | 3.45 | 18.18 | 60 | 42.42 |
| 32 | 8.2 | 21.52 | 12.2 | 46.47 |
| 31 | 16.3 | 60 | 47.84 | 30.1 |
| 46 | 12.09 | 22.91 | 28.02 | 60 |
| 55 | 11.14 | 60 | 60 | 42.08 |
| 28 | 25.1 | 31.81 | 24.35 | 42.4 |

**B**

| Normal | | | |
|---|---|---|---|
| **Veh** | **Dios10** | **Dios20** | **Dios40** |
| 34 | 28.18 | 50 | 50.42 |
| 32 | 30.52 | 22.2 | 46.47 |
| 31 | 45 | 47.84 | 35.1 |
| 46 | 32.91 | 38.02 | 50 |
| 55 | 55 | 50 | 42.08 |
| 28 | 31.81 | 44.35 | 40.4 |

Secondary latency (T2) on passive avoidance memory in shuttle box test in CUMS conditions (**A**) and in non-stressful conditions (**B**). Normal: Healthy group. Veh: Diosmetin solvent received group. Dios: Diosmetin received group (10, 20, 40 mg/kg).

**Supp. Table 2.** The datasets of Novel Object test

**A**

| T2 | | | | |
|---|---|---|---|---|
| CUMS | | | | |
| Normal | Veh | Dios10 | Dios20 | Dios40 |
| 88.11 | 43.89 | 77.32 | 88.11 | 91.16 |
| 63 | 41.68 | 99.11 | 98.21 | 101.13 |
| 66 | 42.77 | 91.18 | 55.39 | 156 |
| 88 | 52.92 | 80 | 88.19 | 139 |
| 73 | 55.89 | 53.19 | 101 | 145 |
| 101 | 38 | 88.17 | 78.21 | 188 |

**B**

| T2 | | | |
|---|---|---|---|
| Normal | | | |
| Veh | Dios10 | Dios20 | Dios40 |
| 88.11 | 102.16 | 102.16 | 102.16 |
| 63 | 115.13 | 121.13 | 121.13 |
| 66 | 116 | 146 | 156 |
| 88 | 139 | 127 | 139 |
| 73 | 105 | 105 | 155 |
| 101 | 88 | 140 | 188 |

**C**

| Recognition Index | | | | |
|---|---|---|---|---|
| CUMS | | | | |
| Normal | Veh | Dios10 | Dios20 | Dios40 |
| 0.590563 | 0.306923 | 0.743462 | 0.778082 | 0.856204 |
| 0.765483 | 0.320615 | 0.883176 | 0.794322 | 0.892507 |
| 0.669794 | 0.279543 | 0.75775 | 0.586138 | 0.917647 |
| 0.594172 | 0.315 | 0.706714 | 0.870497 | 0.91986 |
| 0.52259 | 0.388125 | 0.814548 | 0.892936 | 0.872916 |
| 0.695838 | 0.357716 | 0.844945 | 0.886634 | 0.925561 |

**D**

| Recognition Index | | | |
|---|---|---|---|
| Normal | | | |
| Veh | Dios10 | Dios20 | Dios40 |
| 0.590563 | 0.756204 | 0.806204 | 0.896204 |
| 0.765483 | 0.892507 | 0.812507 | 0.892507 |
| 0.669794 | 0.610647 | 0.717647 | 0.987647 |
| 0.594172 | 0.51986 | 0.91986 | 0.91986 |
| 0.52259 | 0.772916 | 0.772916 | 0.872916 |
| 0.695838 | 0.625561 | 0.655561 | 0.925561 |

The duration of new object recognition (T2) in CUMS condition (**A**) and in stress-free condition (**B**). The datasets of recognition index in CUMS condition (**C**) and in stress-free condition (**D**) in the novel object test. Normal: Healthy group. Veh: Diosmetin solvent received group. Dios: Diosmetin received group (10, 20, 40 mg/kg).

**Supp. Table 3.** The datasets of serum Corticosterone level

**A**

| CUMS | | | | |
|---|---|---|---|---|
| **Normal** | **Veh** | **Dios10** | **Dios20** | **Dios40** |
| 22.49 | 83.17 | 88.95 | 60.18 | 24.33 |
| 12.56 | 93.96 | 73.147 | 55.741 | 28.936 |
| 10.91 | 70.55 | 53.96 | 46.952 | 38.144 |
| 23.002 | 76.542 | 90.341 | 67.531 | 23.982 |
| 27.098 | 52.761 | 64.067 | 41.788 | 58.064 |

**B**

| Normal | | | |
|---|---|---|---|
| **Veh** | **Dios10** | **Dios20** | **Dios40** |
| 22.49 | 16.87 | 14.76 | 4.33 |
| 12.56 | 10.45 | 9.91 | 12.66 |
| 10.91 | 9.22 | 11.61 | 7.91 |
| 23.002 | 12.004 | 8.9311 | 8.428 |
| 27.098 | 12.237 | 11.437 | 4.067 |

Corticosterone level in CUMS condition (**A**) and stress-free conditions (**B**). Normal: Healthy group. Veh: Diosmetin solvent received group. Dios: Diosmetin received group (10, 20, 40 mg/kg).

**Supp. Table 4.** The datasets of Antioxidant Capacity

**A**

| Brain | | | | |
|---|---|---|---|---|
| CUMS | | | | |
| Normal | Veh | Dios10 | Dios20 | Dios40 |
| 356 | 96 | 231 | 259 | 358 |
| 398 | 130 | 251 | 275 | 342 |
| 452 | 88 | 224 | 285 | 333 |
| 402 | 85 | 254 | 241 | 359 |
| 400 | 123 | 201 | 264 | 305 |
| 361 | 62 | 213 | 294 | 330 |
| | 98 | 245 | 278 | 345 |

**B**

| Brain | | | |
|---|---|---|---|
| Normal | | | |
| Veh | Dios10 | Dios20 | Dios40 |
| 356 | 437 | 451 | 546 |
| 398 | 392 | 347 | 592 |
| 452 | 333 | 421 | 603 |
| 402 | 412 | 510 | 512 |
| 400 | 446 | 509 | 589 |
| 361 | 412 | 455 | 491 |
| | 445 | 413 | 472 |

**C**

| Serum | | | | |
|---|---|---|---|---|
| CUMS | | | | |
| Normal | Veh | Dios10 | Dios20 | Dios40 |
| 566 | 231 | 298 | 311 | 451 |
| 575 | 252 | 259 | 351 | 402 |
| 599 | 261 | 301 | 317 | 498 |
| 600 | 270 | 310 | 300 | 431 |
| 581 | 202 | 288 | 341 | 485 |
| 531 | 212 | 265 | 318 | 471 |
| | 215 | 292 | 325 | 432 |

**D**

| Serum | | | |
|---|---|---|---|
| Normal | | | |
| Veh | Dios10 | Dios20 | Dios40 |
| 566 | 689 | 616 | 646 |
| 575 | 598 | 698 | 798 |
| 599 | 561 | 701 | 711 |
| 600 | 750 | 514 | 654 |
| 581 | 680 | 784 | 704 |
| 531 | 501 | 601 | 841 |
| | 575 | 623 | 725 |

Antioxidant capacity of brain tissue in CUMS (**A**), under stress-free conditions (**B**), serum antioxidant capacity CUMS conditions (**C**) and stress-free conditions (**D**). Normal: Healthy group. Veh: Diosmetin solvent received group. Dios: Diosmetin received group (10, 20, 40 mg/kg).

**Supp. Table 5.** The datasets of Malondialdehyde level

**A**

| Brain | | | | |
|---|---|---|---|---|
| CUMS | | | | |
| **Normal** | **Veh** | **Dios10** | **Dios20** | **Dios40** |
| 135 | 400 | 196 | 212 | 195 |
| 165 | 417 | 201 | 228 | 159 |
| 255 | 428 | 236 | 208 | 180 |
| 151 | 458 | 215 | 298 | 161 |
| 188 | 429 | 235 | 224 | 155 |
| 104 | 431 | 210 | 220 | 215 |
| 107 | 418 | 298 | 234 | 199 |

**B**

| Brain | | | |
|---|---|---|---|
| Normal | | | |
| **Veh** | **Dios10** | **Dios20** | **Dios40** |
| 135 | 110 | 98 | 73 |
| 165 | 132 | 118 | 65 |
| 255 | 180 | 105 | 129 |
| 151 | 109 | 201 | 86 |
| 188 | 129 | 162 | 155 |
| 104 | 174 | 115 | 106 |
| 107 | 169 | 123 | 76 |

**C**

| Serum | | | | |
|---|---|---|---|---|
| CUMS | | | | |
| **Normal** | **Veh** | **Dios10** | **Dios20** | **Dios40** |
| 209 | 487 | 199 | 236 | 82 |
| 211 | 463 | 201 | 258 | 114 |
| 185 | 496 | 201 | 206 | 111 |
| 188 | 425 | 245 | 237 | 154 |
| 145 | 479 | 236 | 199 | 89 |
| 188 | 417 | 254 | 211 | 95 |
| | 427 | 301 | 186 | 104 |

**D**

| Serum | | | |
|---|---|---|---|
| Normal | | | |
| **Veh** | **Dios10** | **Dios20** | **Dios40** |
| 209 | 99 | 183 | 82 |
| 211 | 210 | 91 | 114 |
| 185 | 131 | 83 | 91 |
| 188 | 116 | 147 | 89 |
| 145 | 92 | 69 | 72 |
| 188 | 201 | 166 | 95 |
| | 119 | 74 | 104 |

MDA of hippocampal tissue in CUMS condition (**A)**, in stress-free condition (**B**), serum MDA levels in CUMS condition (**C**) and in stress-free condition (**D**).Normal: Healthy group. Veh: Diosmetin solvent received group. Dios: Diosmetin received group (10, 20, 40 mg/kg).
